# Pathways to wheat self-sufficiency in Africa

**DOI:** 10.1016/j.gfs.2023.100684

**Published:** 2023-06

**Authors:** João Vasco Silva, Moti Jaleta, Kindie Tesfaye, Bekele Abeyo, Mina Devkota, Aymen Frija, Innocent Habarurema, Batiseba Tembo, Haithem Bahri, Alaa Mosad, Gerald Blasch, Kai Sonder, Sieglinde Snapp, Frédéric Baudron

**Affiliations:** a International Maize and Wheat Improvement Center (CIMMYT), Harare, Zimbabwe; b International Maize and Wheat Improvement Center (CIMMYT), Addis Ababa, Ethiopia; c International Center for Agricultural Research in the Dry Areas (ICARDA), Rabat, Morocco; d International Center for Agricultural Research in the Dry Areas (ICARDA), Tunis, Tunisia; e Rwanda Agriculture and Animal Resources Development Board (RAB), Rwerere, Rwanda; f Zambia Agricultural Research Institute (ZARI), Lusaka, Zambia; g Institut National de Recherche en Génie Rural Eaux et Forêts (INRGREF), Tunis, Tunisia; h Agriculture Research Center (ARC), Kafr El Sheikh University, Kafr El Sheikh, Egypt; i International Maize and Wheat Improvement Center (CIMMYT), Texcoco, Mexico

**Keywords:** Import substitution, Yield gaps, Genetic improvement, Agronomy

## Abstract

A growing urban population and dietary changes increased wheat import bills in Africa to 9% per year. Though wheat production in the continent has been increasing over the past decades, to varying degrees depending on regions, this has not been commensurate with the rapidly increasing demand for wheat. Analyses of wheat yield gaps show that there is ample opportunity to increase wheat production in Africa through improved genetics and agronomic practices. Doing so would reduce import dependency and increase wheat self-sufficiency at national level in many African countries. In view of the uncertainties revealed by the global COVID-19 pandemic, extreme weather events, and world security issues, national policies in Africa should re-consider the value of self-sufficiency in production of staple food crops, specifically wheat. This is particularly so for areas where water-limited wheat yield gaps can be narrowed through intensification on existing cropland and judicious expansion of rainfed and irrigated wheat areas. Increasing the production of other sources of calories (and proteins) should also be considered to reduce dependency on wheat imports.

## Highlights

• The gap between wheat consumption and production in Africa is increasing over time.
• Wheat yield gaps are large across most wheat producing regions in Africa.
• Improved genetics and agronomy can increase wheat production on existing land.
• Expanding wheat areas is possible with irrigation and drought-tolerant varieties.
• National policies in Africa should re-consider the value of wheat self-sufficiency.

## 1 Introduction

Africa spends 85 billion USD annually on food imports, of which 15% are for wheat imports alone (FAO, 2021). Wheat imports are particularly high for countries in Northern Africa, who are responsible for 59% of Africa's wheat import bill, followed by countries in Western (19%) and Eastern Africa (14%; FAO, 2021). Moreover, Africa's wheat import bill has been increasing over the past two decades at a rate of 9% per year due to population growth, urbanization, and less consumption of coarse grains (Tadesse et al., 2019; Reardon et al., 2021; Noort et al., 2022). Thus, wheat imports have been necessary to fill the widening gap between wheat consumption and wheat production in the continent. However, reliance on imported wheat is becoming a serious challenge considering recent anthropogenic and natural crises disrupting production and trade systems worldwide. Short- and medium-term strategies (Bentley et al., 2022) are needed to prevent over reliance on wheat imports, which could jeopardize food and national security.

Supporting domestic wheat production through better practices and proper technology transfer is indispensable to ensure affordable wheat prices for consumers. In this regard, the experience related to the 2008 wheat price spike shows that most countries across Africa responded to the increased prices with increases in wheat production in the year following the incident (Supplementary Table 1). For instance, compared to 2008, Algeria and Tunisia increased their wheat area in 2009 by 84% and 47%, respectively. In addition to area expansion, remarkable yield improvement was also observed in Northern African countries. Combined effects of area expansion and yield improvement contributed to above 70% of wheat production increment in Algeria, Morocco, and Tunisia. Similarly, Ethiopia, Tanzania, and Zambia expanded the area under wheat production, which resulted into a tangible increase in wheat production in 2009. Alike the 2008 crisis, the ongoing conflict between Russia and Ukraine, the two major wheat exporters, aggravated input prices and food security worldwide (Behnassi and El Haiba, 2022). This calls for designing and implementing initiatives in response to the shortages of wheat stocks in the world market and reducing wheat import bills of nations in Africa through expanding domestic wheat production.

Recent studies concluded that wheat productivity can be increased through narrowing yield gaps on some of Africa's wheat producing areas (Silva et al., 2021a; Baudron et al., 2019; Negassa et al., 2012). The yield gap of irrigated or rainfed crops is defined as the difference between the potential (Yp) or water limited potential yield (Yw) and the actual yield (Ya) realized in farmers' fields, respectively (van Ittersum et al., 2013). Increases in input use combined with good agronomic practices and improved varieties are key to narrow wheat yield gaps in smallholder farming systems in Eastern Africa (Silva et al., 2021a; Baudron et al., 2019) whereas water management is also paramount in Northern Africa (Oweis and Hachum, 2006). Adoption of technologies and functional seed systems and markets are equally important to create a conducive environment for farmers to increase wheat production (Frija et al., 2021a; Shiferaw et al., 2013), which can be fostered through proper policy and institutional innovations.

The objective of this paper was to assess the continental and country level potential to increase wheat production in Africa and to highlight agronomic interventions that can improve wheat yield in the short- and medium-term. We argue that coordinated efforts in disseminating improved varieties and crop management practices, disease monitoring and control, and access to complementary inputs could enhance wheat productivity in Africa, particularly in smallholder farming systems. Identifying areas with high potential for increasing wheat yield, with minimum negative impact on biodiversity, could further help sustaining wheat production in the continent.

## 2 Trends in wheat consumption and self-sufficiency

Cereals cover about half of the energy sources in the daily food consumption in Africa. Among cereals, wheat stands first in terms of per capita consumption, and constitutes about 30% of the cereal consumption in the continent. This goes up to 63% in Northern Africa, where nations heavily depend on wheat consumption for their daily calorie intake, and where most of the wheat is imported (Table 1). Though the per capita consumption of maize is the highest among the cereals consumed in Eastern and Southern Africa, the role of wheat in these regions is also substantial, i.e., between 17 and 32% of the total cereal consumption (FAO, 2021).

**Table 1:** Trends in wheat imports, and respective annual growth rate, across different regions in Africa over the period 1990–2020. Source: FAOSTAT.

Region	Average 2018–2020 (Mt)	Share of each region (%)	Average annual growth rate of wheat quantity import (yr^−1^)		
			1991–2000	2001–2010	2011–2020
Eastern Africa	5.7	12.2	7.9	7.5	2.0
Middle Africa	1.8	3.9	4.4	7.1	7.7
Northern Africa	27.8	59.9	1.9	5.0	2.0
Southern Africa	2.3	5.0	11.9	8.7	4.1
Western Africa	8.9	19.1	12.1	6.3	5.1
**Africa (Total)**	**46.5**	**100.0**	**3.6**	**5.5**	**2.7**

**Fig. 1:**
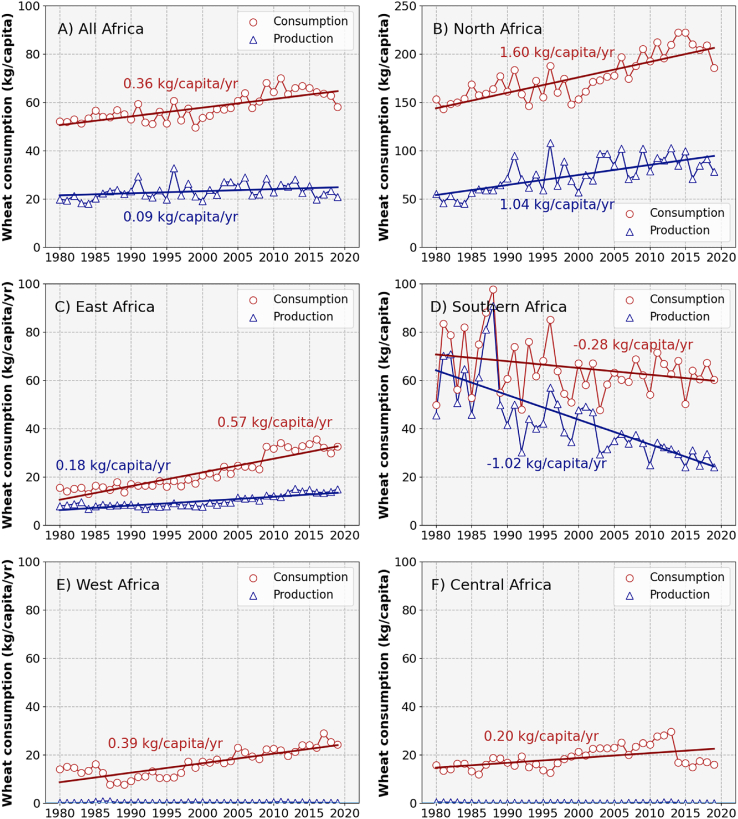
Historical trends in wheat consumption per capita met by production and imports across all of Africa (A), Northern Africa (B), East Africa (C), Southern Africa (D), West Africa (E), and Central Africa (F). Regression lines were fitted to the data, and respective slopes are displayed in each panel. Note differences in the scale of the y-axis between (B) and the other panels in the figure. Source: FAOSTAT.

Wheat consumption across Africa was ca. 60 kg capita^−1^ in 2019 and increased over time at a rate of 0.36 kg capita^−1^ yr^−1^ (Fig. 1A). Conversely, domestic wheat production would only sustain a wheat consumption level of ca. 20 kg capita^−1^ across Africa, a value that has been stable during the past four decades. Wheat consumption has historically been highest in Northern Africa (Fig. 1B), intermediate in East and Southern Africa (Fig. 1C–D), and lowest in West and Central Africa (Fig. 1E–F). Yet, the gap between production and consumption has widened over the past four decades in all five regions (Fig. 1). In Northern Africa, per capita wheat consumption increased linearly over the past four decades to nearly 200 kg capita^−1^ in the most recent years, with domestic production currently satisfying slightly less than half of that demand (Fig. 1B). Wheat demand in East Africa also increased linearly over the past four decades, but at a slower pace than in Northern Africa (0.57 vs. 1.60 kg capita^−1^ yr^−1^; Fig. 1B–C). Current wheat consumption levels in East Africa reach ca. 30 kg capita^−1^ yr^−1^, half of which can be satisfied with domestic production (Fig. 1C). In contrast with the other regions, wheat consumption slightly declined in Southern Africa, at a rate of 0.28 kg capita^−1^ yr^−1^, reaching a level of 60 kg capita^−1^ in 2019 (Fig. 1D). Domestic production in Southern Africa satisfied nearly all wheat demand in the early 1980s, but only about a third since 2015. Lastly, wheat consumption in West and Central Africa was about 20 kg capita^−1^ in 2019 and increased at a rate of 0.39 and 0.20 kg capita^−1^ yr^−1^, respectively (Fig. 1E–F). All the remaining wheat demand in these regions has been met with imports.

Foresight studies show that per capita wheat consumption in Middle East and Northern African countries is not expected to further increase by 2050. The growth of wheat demand in these regions is predicted to be driven by population growth. Per capita wheat consumption is however expected to increase in many Asian and sub-Saharan African countries (Frija et al., 2021b).

**Fig. 2:**
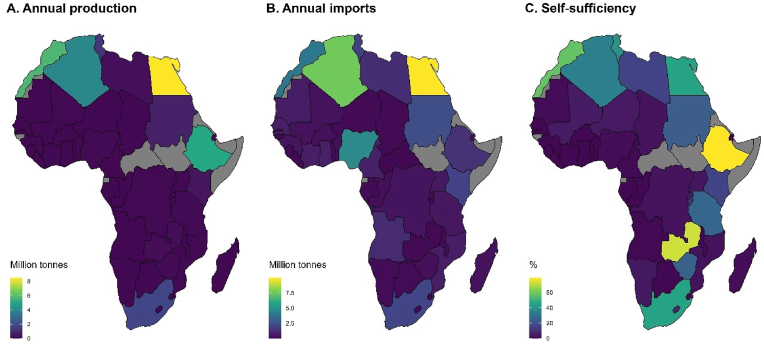
Annual wheat production by country (A), annual wheat imports by country (B), and wheat self-sufficiency by country (C) in Africa. Data refer to averages over two years, 2018–2019. Source: FAOSTAT.

Wheat self-sufficiency ratios at national level differ considerably across Africa (Fig. 2C). For instance, Ethiopia reached ca. 80% wheat self-sufficiency in 2018 and 2019 because of increased wheat production. Zambia was also able to reach a high degree of self-sufficiency in wheat (73%), the second highest in the continent after Ethiopia, over the same period due to low consumption levels. Conversely, domestic wheat production in Egypt only met 45% of the country's wheat demand in 2018 and 2019, which is a result of high wheat consumption met through high wheat imports. Domestic production in all other countries in Northern, Eastern, and Southern Africa satisfied 10–40% of their wheat consumption. When food crises occur, with associated spikes in prices of imported food commodities, consumed cereals might be expected to shift to favor locally produced over imported ones. Yet, this has not been observed for the major wheat producing countries in Africa (Supplementary Table 1).

## 3 Trends in wheat production, harvested area, and actual yield

**Fig. 3:**
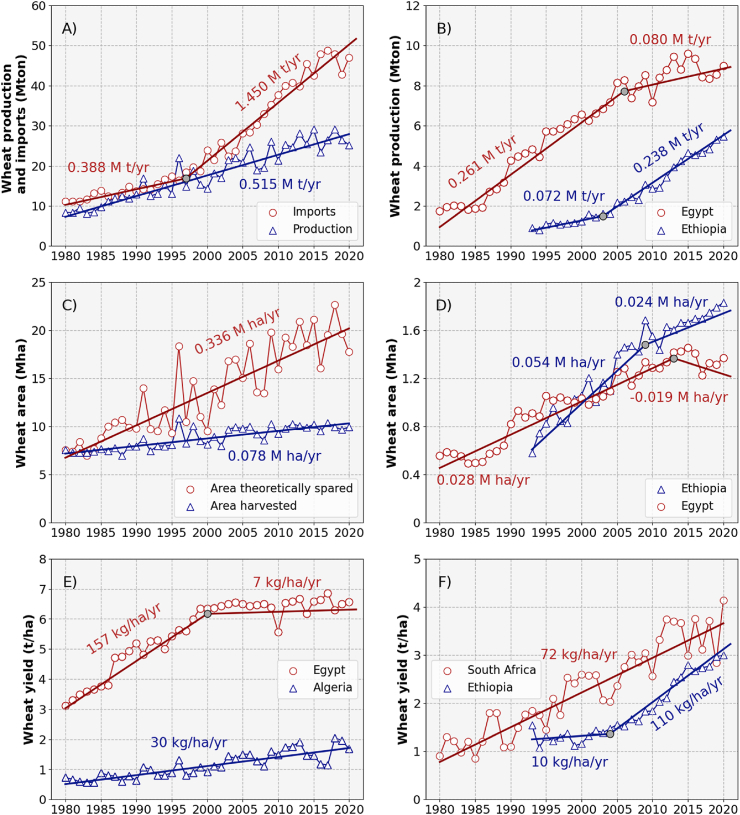
Historical trends in wheat production (tons), harvested area (ha), and yield (t ha^−1^) across Africa and for the main wheat producing countries in the region (in terms of harvested area). The area theoretically spared in panel (C) was estimated as the additional land that would have been required to reach the amount of wheat produced in a particular year if yields had remained at their 1980 levels. Segmented regression lines were fitted to the data, and respective slopes are displayed in each panel. Source: FAOSTAT.

Wheat production in Africa increased steadily at a rate of 0.52 million tons per year (Mt yr^−1^) since 1980 to nearly 25.2 Mt in 2020 (Fig. 3A). Such increase in wheat production was accompanied by a much sharper increase in wheat imports: since the mid-1990s wheat imports increased steadily at a rate of 1.45 Mt yr^−1^, resulting in a gap between wheat import and production of 21.7 Mt in 2020. The magnitude of the increase in wheat production varied across countries (Fig. 3B). For instance, Ethiopia (the largest wheat producer in Eastern Africa) saw spectacular increases in wheat production, at a rate of 0.24 Mt yr^−1^, since the early 2000s. Conversely, Egypt (the largest wheat producer in Northern Africa) has experienced negligible increases in wheat production during the same period, following three decades of steady increases at a rate of 0.26 Mt yr^−1^.

Increases in wheat production across Africa were accompanied by modest increases in wheat harvested areas (Fig. 3C). About 10 Mha of wheat were harvested across the continent in 2020, an increase of ca. 2.5 Mha since 1980. From a theoretical point of view, increased wheat yield led to the sparing of an estimated 0.34 Mha of land every year, land that would have been put under wheat cultivation to maintain actual production if yields had remained at their 1980 levels (Fig. 3C). Trends in wheat area were also different across countries (Frija et al., 2021a), but it is remarkable that the rate of wheat area expansion slowed down since ca. 2010 in countries like Ethiopia and Egypt (Fig. 3D). Ethiopia reported a wheat harvested area of 1.83 Mha in 2020. Prior to that, the country experienced increases in wheat area of 0.05 Mha yr^-1^ until 2010, followed by an expansion of the wheat area at about half of that rate since then. A more extreme case was observed in Egypt, a country with nearly 1.2 Mha of wheat harvested in 2020, where slight decreases in wheat area were observed since ca. 2010 after three decades of wheat area expansion at a rate of 0.03 Mha yr^−1^.

The top four wheat producing countries in Africa experienced a mix of yield advances (e.g., Algeria, Ethiopia, and South Africa) and yield plateau over the past four decades (i.e., Egypt; Fig. 3E–F). Wheat yield was lowest in Algeria, where wheat yield progress has also been lowest, 30 kg ha^−1^ yr^−1^, corresponding to a yield increase from 0.7 to 1.7 t ha^−1^ between 1980 and 2020 (Fig. 3E). Wheat yield progress in South Africa was also linear, 72 kg ha^−1^ yr^−1^, corresponding to an increase in wheat yield from 0.9 t ha^−1^ in 1980 to 4.1 t ha^−1^ in 2020 (Fig. 3F). In Ethiopia, wheat yield increased following a linear piecewise trendline with increasing rate over time (Fig. 3E). Small rates of yield progress, 10 kg ha^−1^ yr^−1^, were observed until 2005, with average wheat yields of ca. 1.2 t ha^−1^. Since then, wheat yield increased at a rate of 110 kg ha^−1^ yr^−1^, reaching a maximum of 3.0 t ha^−1^ in 2020. Lastly, wheat yield progress in Egypt was described by a linear trend with an upper plateau after the year 2000 (Fig. 3E). The upper plateau corresponds to 6.6 t ha^−1^ and follows a period with increases in wheat yield of nearly 160 kg ha^−1^ yr^−1^. The scope to increase wheat yield in the future is thus country specific (Frija et al., 2021a), but analysis of yield gaps is needed to seize the extra production that can be realized in each country on existing wheat areas (see Section 5.1).

## 4 Wheat trade with Africa and world market prices

**Fig. 4:**
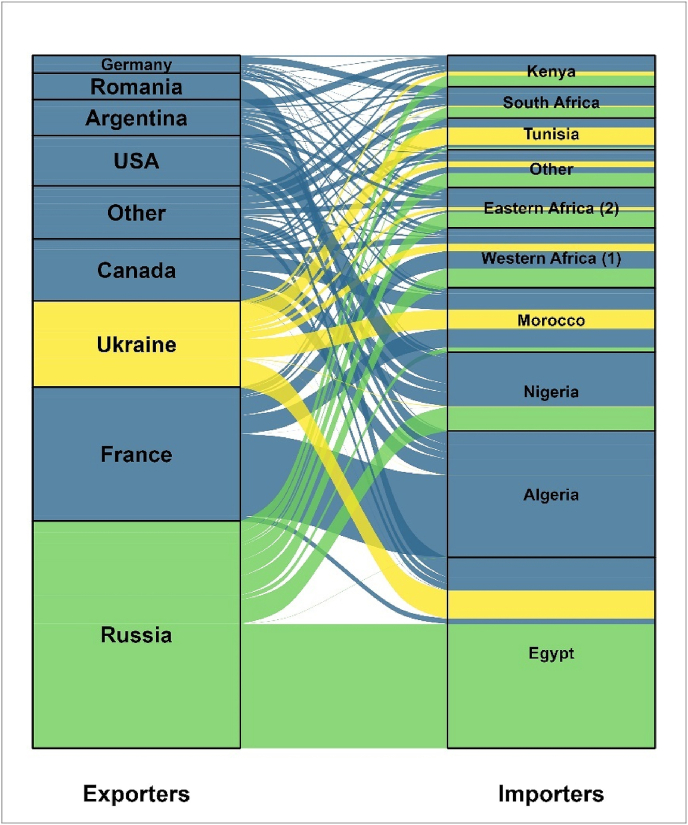
Wheat trade with Africa in the period 2018–2019. The height of a block represents the volume of wheat exported/imported, and the height of a stream field represents the volume of wheat traded by the two countries connected by the stream field. Codes: (1) excluding Nigeria, (2) excluding Kenya. Source: FAOSTAT.

In 2020, Africa imported 46.5 million tons of wheat grain corresponding to nearly 25% of the global wheat imports (FAO, 2021). As the major wheat exporting countries are limited in number, global wheat trade is highly concentrated. A high trade concentration ratio (i.e., trading a large proportion of specific commodities with a limited number of countries) could make countries vulnerable to unexpected shocks deterring trade in specific regions. Extreme weather events, disease outbreaks, or conflicts in exporting countries are good examples of unexpected shocks affecting trade. Wheat imports in Africa showed a high trade concentration ratio as 60–70% of the wheat imported to the continent originated from six countries, namely Russia, France, Ukraine, USA, Canada, and Argentina (Fig. 4). The trade concentration ratio was even worse for individual countries. For instance, more than 60% of the wheat imports in Egypt and Algeria, the largest wheat importing countries in Africa during 2018 and 2019, originated from a single country: Russia for Egypt and France for Algeria (Fig. 4). Such heavy dependence on few countries as a source of import makes adjustment to shocks challenging, as observed with the recent Russia-Ukraine conflict.

**Fig. 5:**
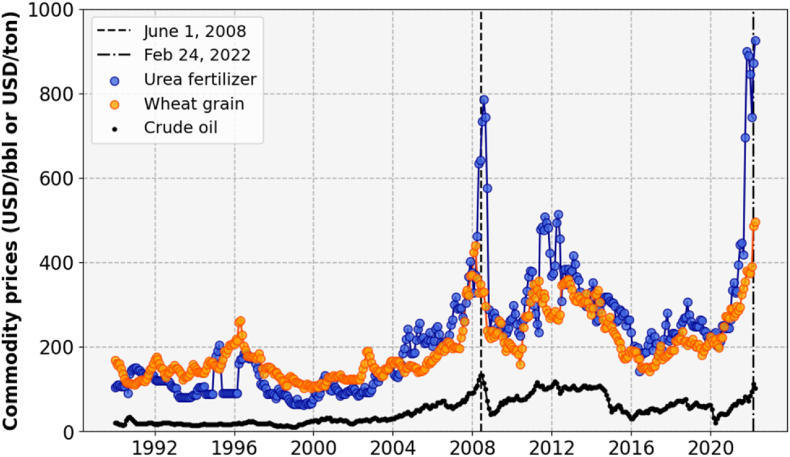
Trends in world market prices for wheat grain, urea fertilizer, and crude oil over the period 1990–2022. Vertical lines indicate June 1, 2008 (when world market prices spiked) and February 24, 2022 (marking the start of Russia-Ukraine conflict). Source: World Bank Commodity Price.

The world market price of wheat is highly volatile (Guo and Tanaka, 2019) and usually connected to fuel prices, i.e., the main source of energy for farm operations and production of nitrogen (N) fertilizers. The wheat price was particularly high in the past two decades during the 2007/8 economic crises, when high fuel prices were observed in 2011 and 2013, and at the start of the Russia-Ukraine conflict in 2022 (Fig. 5). Increases in the world market price of wheat put wheat importing countries under heavy import bills, which can have severe effects when it occurs suddenly. Such a price hike in the world market is quickly transmitted to domestic markets, where it often stimulates governments and farmers to boost domestic production.

## 5 Opportunities to increase wheat yield in the short- to medium-term

### 5.1 Wheat yield gaps in Africa

**Fig. 6:**
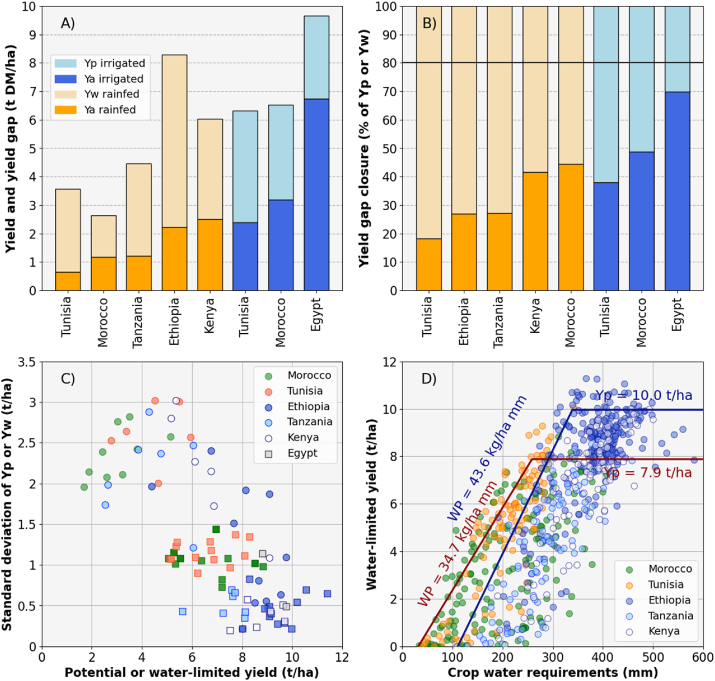
Yield and yield gaps (A and B), yield variability (C), and water productivity (D) for irrigated and rainfed wheat in the main producing countries of Northern and Eastern Africa. Deviations from the boundary line in (D) indicate yield gaps due to sub-optimal distribution of rainfall in specific growing seasons (see also Rattalino Edreira et al., 2018). Two boundary lines were fitted in (D) to acknowledge different wheat mega-environments between Eastern and Northern Africa (Negassa et al., 2012). Eastern Africa falls under mega-environment 2, which is characterized by “high rainfall in summer; wettest quarter mean minimum temperature >3 °C and <16 °C, wettest quarter (three consecutive wettest months) precipitation >250 mm; elevation 1400 m; with spring wheat growth habit”. By contrast, Northern Africa falls under mega-environment 9, which is characterized by “low rainfall <400 mm, winter/spring rainfall dominant with facultative wheat growth habit”. Source: Global Yield Gap Atlas (www.yieldgap.org) and van Ittersum et al. (2016).

Opportunities to increase wheat yield depend on the magnitude of existing yield gaps, and on the interventions available to narrow them. Wheat yield gaps are large for most countries in Africa, with values above 50% of Yp or Yw (Fig. 6A and B; van Ittersum et al., 2016 as available in www.yieldgap.org). The exception is Egypt, where the yield gap for irrigated wheat is ca. 30% of Yp, corresponding to a yield gap of ca. 1 t ha^−1^.

Wheat is cultivated under rainfed and irrigated conditions in Northern Africa, whereas wheat production in Eastern Africa (Ethiopia, Kenya, and Tanzania) is predominantly rainfed (www.yieldgap.org). Wheat Ya in Morocco was on average 1.2 t ha^−1^, about half of what could be achieved with good agronomic practices (Yw of 2.6 t ha^−1^). In Tunisia, wheat yield under rainfed conditions reached less than 20% of Yw (0.5 t ha^−1^ against Yw of 3.5 t ha^−1^) and under irrigated conditions reached close to 40% of Yp (2.5 t ha^−1^ against Yp of 6.5 t ha^−1^). Similarly large yield gaps were found for rainfed wheat in East African countries (see also van Ittersum et al., 2016). In Ethiopia, wheat yield reached only ca. 25% of Yw, corresponding to Ya of 2.3 t ha^−1^ and Yw of 8.2 t ha^−1^ (Silva et al., 2021a). Wheat yield was also only ca. 25% of Yw in Tanzania, although Yw in Tanzania was about half of the Yw in Ethiopia. In Kenya, wheat yield reached ca. 40% of Yw, which was on average 6 t ha^−1^. In summary, wheat yield gaps are large in Northern and Eastern Africa, indicating it is agronomically possible to more than double wheat yields across the main producing areas in both regions.

Narrowing yield gaps entails risks and costs for farmers and can thus be more achievable in the short-to medium-term in some regions than in others. Variability in Yw over time is a good indicator of climatic risk due to inter-annual fluctuations in soil water availability. Irrigated wheat can deliver high and stable yields over time, whereas rainfed wheat suffers from high inter-annual yield variability (except few areas in the Ethiopian highlands, Fig. 6C). Large fluctuations in crop yield make it difficult for farmers to invest in capital intensive inputs as returns do not materialize under risky, water limited conditions. Farmers operating in such environments often favor technologies that are less risky, and potentially less profitable (Hansen et al., 2004).

Input-output price ratios largely determine if narrowing yield gaps is profitable from an economic perspective (Bonilla-Cedrez et al., 2021). Many African countries could increase wheat production profitably under existing input-output price ratios and moderate levels of fertilizer use. For instance, Ethiopia could produce more than 9 Mt of wheat profitably with existing market prices and 50% of the recommended fertilizer rate (Negassa et al., 2012). African countries could also increase wheat production through expanding areas under irrigated wheat and improve irrigated wheat yield with improved crop management practices. Such practices include adjustments in planting dates to escape extreme growing-season temperature (Xiao et al., 2017), integrated and site-specific nutrient management (Darjee et al., 2022; Abhijit et al., 2007; Khurana et al., 2008) , and proper mechanization in irrigated wheat production (Banaeian and Zangeneh, 2011).

### 5.2 Wheat improvement and seed systems

Many wheat varieties have been released across Africa depending on government's attention to wheat as a national strategic food security crop. For example, 173 bread and durum wheat varieties were released in Ethiopia since 1967 though not more than four dozen were grown each year (MoA, 2020). Many varieties became obsolete due to new rust races, low yields, slow early generation seed multiplication, and weak extension systems in promoting and scaling newly released wheat varieties. In South Africa, 291 varieties were released between 1891 and 2013 (Nhemachena and Kirsten, 2017). In Morocco, 171 bread and durum wheat varieties were released between 1982 and 2012 (Andalousi et al., 2019). By contrast, only 16 wheat varieties were released in Nigeria since 1965 (Nigerian Seed Portal Initiative).

Yield potential and disease resistance are important traits for which modern wheat varieties have been bred for. Genetic gain in yield potential is routinely measured in breeding programs, though its value depends on the germplasm used and the testing method deployed. For instance, analysis of national performance trials (194 advanced lines) across 20 sites in Ethiopia indicated a wheat genetic gain of 0.94% yr^−1^ between 2014 and 2019. The estimated genetic gain for irrigated wheat in South Africa, between 1998 and 2013, was 0.82% yr^−1^ in the Eastern Highveld and 0.40% yr^−1^ in the cooler Central areas, whereas a limited gain was observed in the warmer Northern areas and KwaZulu-Natal province (Dube et al., 2019). In Morocco, field trials in six sites of twenty-nine durum cultivars revealed a genetic gain of 0.43% yr^−1^ (1949–2016), mostly associated with selection for early flowering and higher harvest index (Taghouti et al., 2014). Modern wheat varieties also include gains in heat tolerance allowing to grow wheat at lower elevations in a broader range of cropping systems in tropical Africa (Pequeno et al., 2021).

New varieties are the backbone of a robust seed system. Wheat varieties are being developed and released in Africa. Yet, farmers’ access to improved varieties remains a major challenge (Tadesse et al., 2019; Frija et al., 2021a). Wheat seed systems in Africa comprise both formal and informal exchanges of seed, with informal seed systems (i.e., farmer to farmer exchange) being dominant in most countries (Bishaw et al., 2010; McGuire and Sperling, 2016). Limited availability of early generation seed is the main bottleneck facing the formal seed system, whereas seed quality and availability remains the main challenge in the informal seed system (Tadesse et al., 2019). The informal seed system provides seeds of locally adapted cultivars to nearby farmers though it lacks standard certification, and basic infrastructures.

The formal seed sector alone can't supply the desired quality and quantity of seeds to wheat farmers. Early generation seed multiplication must be carried out at scale to deliver seeds of new varieties to farmers quickly. Therefore, for wheat seed to reach farmers, and support productivity gains, it is crucial to (1) strengthen variety development, release, and registration, (2) improve the delivery of early generation seed, (3) strengthen the capacity of public and private sectors to produce large volume of certified seed, (4) develop a reliable seed demand/supply system, and (5) establish a more efficient quality assurance and certification system. Informal seed systems would also benefit from enhanced resilience of community-based seed systems (Mulesa et al., 2021; McGuire and Sperling, 2016) and from technical support on on-farm seed selection, cleaning, separate storage, and related seed management practices that ensure seed quality at household level (Bishaw et al., 2010).

### 5.3 Crop establishment and mechanization

Land preparation and sowing are the most labor-intensive operations for smallholder wheat production (Baudron et al., 2019) and labor constraints during these operations can lead to poor yields (Silva et al., 2019). Appropriate-scale farm mechanization, including the use of low powered, affordable, and easy to maintain two-wheel tractors (Baudron et al., 2015), in combination with direct seeding, can help overcoming labor constraints during peak periods while increasing wheat yield.

**Fig. 7:**
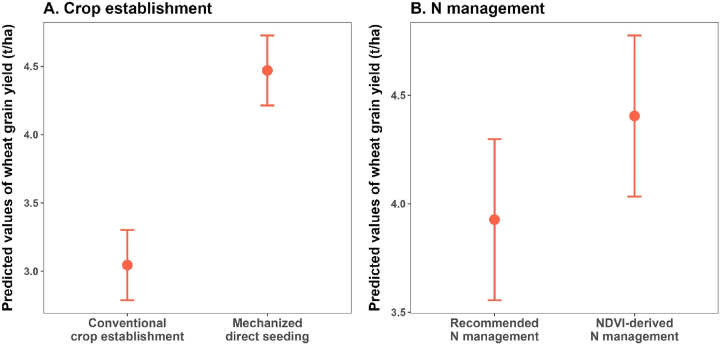
Wheat yield response to improved agronomic practices in Eastern Africa: (A) predicted values of wheat grain yield obtained on-farm in the Southern Highlands of Ethiopia using conventional crop establishment vs. mechanized direct seeding and (B) predicted values of wheat grain yield obtained on-farm in Northern Rwanda using recommended nitrogen (N) management (rate and timing) vs. N management based on readings from a hand-held NDVI sensor at first node stage and booting stage after a basal application. See text for further information.

On-farm trials in the Southern Highlands of Ethiopia revealed that mechanized direct seeding and basal fertilization (in a single operation) using a two-wheel tractor increased wheat yield significantly, by 1.4 t/ha (47%) on average, compared to ‘conventional’ crop establishment (ploughing using animal traction, followed by manual seeding and manual application of basal fertilizer; Fig. 7A). Field observations indicated that higher wheat yields under mechanized direct seeding were the result of better placement of seeds and fertilizer, as well as higher plant population. Plant population is indeed a major determinant of wheat yield gaps in Eastern Africa (Baudron et al., 2019; Silva et al., 2021a). Another study on mechanized wheat establishment also found labor requirements for land preparation and seeding wheat to be 10-fold lower with mechanized direct seeding than with the traditional method. This can lead to greater timeliness of seeding under on-farm conditions, with a positive impact on yield as planting time is often another major determinant of wheat yield gaps in the region (Baudron et al., 2019).

Farm operations for wheat cultivation are mostly mechanized in Northern Africa. On-station experiments and farmer's field surveys confirmed that direct seeding increased wheat yield by 10–20% relative to the ‘conventional’ method of crop establishment (Devkota et al., 2022; Mrabet et al., 2021). Indeed, adoption of direct seeding was shown to be more profitable and less susceptible to risk than practices involving tillage before seeding in Morocco and hence, an important intervention to reduce yield gaps for rainfed wheat (Devkota and Yigezu, 2020). Direct seeding is known to increase the uptake, conservation, and use of available soil water in the Maghreb region (Mrabet et al., 2021) and in the Middle East and Northern Africa region (Devkota et al., 2022). Yet, most direct seeders are expensive and beyond the reach of smallholders. Availability of low-cost direct seeders thus remains important for wider dissemination of direct seeding of wheat in Northern Africa (Devkota et al., 2021, 2022).

### 5.4 Water and nutrient management

Benchmarks for water productivity can be derived using boundary functions between crop yield and water requirements (Fig. 6D; Rattalino-Edreira et al., 2018). The parameters of such boundary functions are biophysically meaningful (Rattalino-Edreira et al., 2018; French and Schulz, 1987): the x-intercept indicates the seasonal soil evaporation, the slope indicates the maximum water productivity, and the plateau indicates the maximum yield obtained without water limitations. The slope and x-intercept for wheat crops in Eastern Africa had a value of 43.6 kg ha^−1^ mm^−1^ and ca. 100 mm, respectively. Slightly smaller values were observed for wheat crops in Northern Africa for which the slope of the boundary line was 34.7 ha^−1^ mm^−1^ and the x-intercept ca. 40 mm. Thus, rainfed wheat crops can produce higher yields per mm of water in Eastern Africa compared to Northern Africa, where judicious use of water is particularly important (Chebil et al., 2016; Oweis and Hachum, 2006). Finally, no further yield increases were observed for water supply above 325 and 270 mm of plant available water meaning such levels of water supply can satisfy crop water requirements to reach a Yp of 10.0 and 7.9 t ha^−1^ in Eastern and Northern Africa, respectively.

Soil fertility and nutrient management are important to consider in an integrated manner. N is the most important determinant of wheat productivity in Africa, where moisture is sufficient (e.g., Silva et al., 2021a; Chebil et al., 2016), and a key driver of wheat protein (an important quality trait). Extension advisories that support good agronomic practices and targeting of N to wheat growth are effective to increase wheat production, and N-use efficiency. For instance, in Northern Rwanda (Baudron et al., 2019), the relationship between NDVI, measured with a hand-held sensor at first node and booting stages, and grain yield was established to generate site-specific recommendations of N rates to apply during these two critical stages, following the basal application of a modest N rate. Results from on-farm trials conducted over three growing seasons revealed that NDVI-derived N management (i.e., 18 kg ha^−1^ of N applied as basal, variable N rates based on NDVI reading applied at first node and booting stages, making a total of 18–105 kg N ha^−1^) increased wheat yield significantly compared to the recommended N management (18 kg N ha^−1^ applied as basal and 23 kg N ha^−1^ applied as top dressing; Fig. 7A). Mean wheat yields in the area range between 3.0 and 3.5 t ha^−1^ (Baudron et al., 2019) and benefit from residual effects of fertilization applied to potato, which receives high rates of inorganic and organic fertilizer. In general, wheat yield response to N can be optimized through multiple split applications where higher amounts are applied in good rainfall years.

Negative N balances were found for wheat in Ethiopia indicating that N application rates are insufficient to maintain current yields (Silva et al., 2021b). One of the key questions in wheat agronomy is thus how to use effectively and profitably the N available through mineral and organic fertilizers. Landscape position was also identified as an important driver of nutrient-use efficiency in the Ethiopian highlands. Results from on-farm trials revealed that fertilizer should be targeted to high responsive sites in micro-topography, as there is a modest crop fertilizer response on steep slopes due to low soil fertility on these landscape positions, with limited soil organic carbon, clay content, and soil water content (Amede et al., 2020).

Wheat yield response to N in the Mediterranean drylands, including Morocco, was noticeable under both low and high yielding conditions (Savin et al., 2020). Indeed, fertilizer management was the second most important variable explaining wheat yield variability in Morocco (Devkota and Yigezu, 2020). Application rates greater than 20 kg N and P ha^−1^ were more profitable and less subject to risk than application rates lower than 20 kg N and P ha^−1^ under rainfed conditions. Under irrigated conditions, N and P application rates ranged between 65 and 120 kg N ha^−1^ and 28–80 kg P ha^−1^. N management of wheat cropping systems in semi-arid areas also depends on the crop rotation adopted. For instance, on-farm trials conducted across four different regions of Tunisia found that N capture by durum wheat was significantly affected by the preceding crop (Ben Zekri et al., 2019). The highest N uptakes of irrigated and rainfed wheat were observed after vegetables, and after legumes, respectively, and the lowest N uptake was observed in cereal-wheat rotations.

### 5.5 Early warning systems for disease monitoring

Early warning systems of disease outbreaks are important to minimize the impact of pests and diseases on wheat production. The Ethiopian wheat rust Early Warning and Advisory System (EWAS) is an example of near real-time predictive capacity within the growing season (Allen-Sader et al., 2019). The EWAS provides daily automated 7-day rust risk forecasts and advisories across Ethiopian wheat production regions in collaboration with partners in the country. The 7-day advanced forecasts for rust risk, combined with approximately 2 weeks for the disease to develop on crops, provide farmers and extension officers up to three weeks warning of wheat rust appearance in a new area, which is deemed sufficient for timely control.

Since its piloting in 2015–2016, the EWAS has been fully operational reaching hundreds of thousands of smallholders. Mottaleb et al. (2021) found a positive benefit from the EWAS in Ethiopia using household surveys and key informant interviews. Changes in farmer behavior (fungicide use, increased awareness on rusts, and rust control) and policy change (reserve stocks of fungicide and a dedicated desk at national bank for fungicide imports) were reported. The EWAS also improved collaboration and coordination at national level through the implementation of rust planning meetings.

In the 2021/22 wheat growing season, there was a very high risk of a major stripe rust outbreak in Ethiopia (CIMMYT, 2022). Yet, thanks to early warning, high levels of engagement with national partners, and preparedness/timely response from officials and farmers, effective control was achieved in many wheat producing areas. Initial forecasts indicate a potential record wheat production with minimal levels of disease and small yield losses (USDA United States Department of Agriculture, 2022). An impact assessment of the EWAS is on-going and the system is currently being adapted to the Kenyan and Zambian wheat disease-production context, where it is expected to be released in 2023.

### 5.6 Wheat area expansion across Africa

**Fig. 8:**
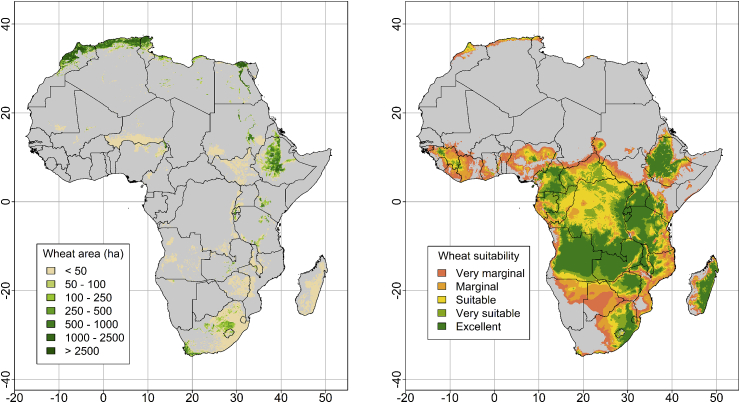
Current wheat cultivated area (left) and wheat area suitability (right) across Africa. Wheat suitability was determined with EcoCrop. Source: Yu et al. (2020) and Negassa et al. (2012).

Wheat area expansion is also a possible pathway to narrow the gap between wheat demand and wheat production in Africa. Wheat suitability mapping indicates that close to 86 Mha of cropland (excluding forests and protected areas) could be suitable for wheat production (Fig. 8; Negassa et al., 2012). This is a much larger area than the ca. 10 Mha sown to wheat in 2020 (Fig. 1C). Throughout Africa, wheat harvested areas are much smaller than areas with high suitability (Supplementary Table 2). In all cases, wheat area expansion needs to be carefully planned to avoid deforestation, undesirable land-use changes, and reductions of existing crop diversity. Diversification is a vital component of sustainable agricultural development, and wheat expansion could endanger this through land conversion.

Considering medium levels of intensification and profitability, twelve countries in sub-Saharan Africa (Angola, Ethiopia, Kenya, Madagascar, Tanzania, DRC, Rwanda, Burundi, Uganda, South Africa, Zambia, and Zimbabwe) could produce wheat profitably on ca. 25 Mha without irrigation (Negassa et al., 2012). The amount of wheat that could potentially be produced by cultivating suitable land for wheat would exceed the current consumption levels. In addition to rainfed wheat production, Africa has more than 23 Mha of land that could be irrigated (You et al., 2010). If wheat would be grown as a double crop on at least half of the potential irrigation area, this could provide an additional 12 Mha for irrigated wheat in the continent.

Irrigated wheat can be grown in lowland areas as a double crop in rotation with other crops. For example, in the irrigated lowlands of Ethiopia, double cropping is practiced by growing wheat after cotton, rice, soybean, or sesame in the winter season (Tadesse et al., 2019, 2022). In the irrigated highland areas of Ethiopia, wheat is often double cropped with maize, potato, or horticultural crops in the off-season, a time when temperatures are conducive for irrigated wheat production. In Sudan, irrigated wheat is rotated with cotton, groundnut, or fodder crops within a season (Ishag, 2015). This is possible due to the availability of improved wheat varieties that include gains in heat tolerance allowing to grow wheat at lower elevations in a broader range of cropping systems (Pequeno et al., 2021; Iizumi et al., 2021). Opportunities for intensification at cropping systems level, with wheat as a double crop, need to be further explored in other lowland regions across Africa.

Wheat is also a good rotation crop in the existing wheat growing areas as it helps conserve soil resources, protects water quality, and breaks the cycle of pests and diseases of non-cereal crops (Snapp et al., 2010). In South Africa, wheat is double cropped with soybean, maize, tobacco, and sunflower depending on soil conditions (Maas and Kotzé, 1990). The use of legumes such as field bean, faba bean, and chickpea in smallholder wheat-based cropping systems was found to be useful to increase wheat yield while reducing fertilizer rates and input costs for smallholders (Tadesse et al., 2019). Rotation of wheat with oil crops such as canola, rapeseed, sesame, and sunflower is also important in major wheat growing areas of Africa as such crops help in weed, pest and disease control, and improve soil structure thanks to their deep tap roots (Tadesse et al., 2019).

## 6 Alternatives to diversify calory and protein sources in Africa

**Fig. 9:**
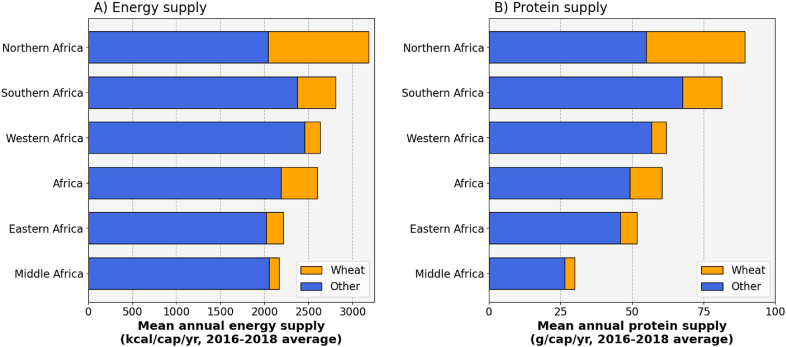
Energy (A) and protein (B) supplied by wheat and other (non-wheat) food sources in Africa and by sub-regions. Source: FAOSTAT.

Wheat represents a major source of energy and protein in African diets. Across the continent, wheat provides an average energy supply of 415 kcal capita^−1^ yr^−1^ (or 16% of the total energy supply, Fig. 9A) and an average protein supply of 11 g capita^−1^ yr^−1^ (or 19% of the total protein supply, Fig. 9B). Yet, there are strike regional differences across Africa (Fig. 9). Energy and protein supply from wheat are highest in Northern Africa, corresponding to 1140 kcal capita^−1^ yr^−1^ (or 36% of the total energy supply) and 35 g capita^−1^ yr^−1^ (or 39% of the total protein supply), and negligible in Central Africa.

Wheat flour is unique in forming a viscoelastic dough that retains gas and sets during baking (Hoseney and Rogers, 1990) and can thus not be substituted for e.g., bread-making. However, flour blends which incorporate flour from other crops, often indigenous, such as cassava or legumes, have been developed to manufacture several wheat-based products (Owusu et al., 2017; Monnet et al., 2019; Noort et al., 2022). Though this tends to modify the texture and taste of these products (Monne et al. 2019), it could significantly reduce wheat imports in the region, with the potential additional benefit of improving the nutritional value of these products (Owusu et al., 2017, Monnet et al., 2019).

Despite the unique technological properties of wheat flour mentioned above, it should be highlighted that the calories and proteins currently supplied by wheat could be replaced to some extent by other commodities whose local production has been increasing on the continent (Supplementary Fig. 1). For instance, the production per capita of roots and tubers has increased substantially in Africa, and particularly so in Northern and Western Africa, over the past six decades, and could be considered as alternative dietary energy sources in a context of wheat-related food shocks. Similarly, the production of pulses per capita, particularly in West Africa, and the production of egg per capita, particularly in Northern Africa, Southern Africa, and West Africa, increased substantially over the past six decades and should be given consideration as substitutes to the proteins currently supplied by wheat.

## 7 Conclusion

Domestic wheat production can be increased in Africa by allocating resources and setting proper policies that support a research and development (R&D) agenda addressing the major bottlenecks affecting current regional wheat production. Three pathways can support such strategy. Firstly, narrowing yield gaps through improved seed systems and agronomy can more than double current wheat yields in rainfed cropping systems. This requires functional and inclusive seed systems and the availability of inputs to farmers in the right volume, at the right cost, and at the right time. Secondly, cropping systems can be further intensified in some regions by adding wheat as a double crop, as demonstrated in irrigated wheat production systems in South Africa, Ethiopia, Sudan, Egypt, and Morocco, hence improving water use and management capacity throughout the year. Finally, the advent of heat-tolerant varieties makes it possible to cultivate wheat under irrigated conditions in lowland areas of the continent. However, attention needs to be paid to protection of natural areas and agriculturally diverse production systems, so that wheat self-sufficiency in Africa is achieved in a sustainable way.

Many African countries have similar environmental and socio-economic constraints to wheat production. Coordinated efforts towards wheat self-sufficiency in the region are possible particularly on trans-boundary constraints (e.g., wheat rusts) and exchange of germplasm. Extension is needed as well, to support the improved agronomy necessary to increase wheat production in the continent. Identification and scaling of context-specific and appropriate mechanization technologies could ensure proper and timely crop establishment and enhance the efficiency in water and fertilizer use to narrow yield gaps. Further attention should also be paid to understand how different technologies should be combined to ensure maximum adoption and to develop farm advisories that can facilitate site-specific crop management. Exploring policy, institutional, and market related gaps influencing the profitability of wheat production and trading across regions in the continent is another key area for further research. Such strategies would help reducing wheat import dependency in many African countries, which should re-consider the value of wheat self-sufficiency as a strategic investment for national economies. Increasing the production of other sources of calories and proteins could also be considered to reduce dependency on wheat imports.

## Declaration of competing interest

The authors declare that they have no known competing financial interests or personal relationships that could have appeared to influence the work reported in this paper.

## Data Availability

Data will be made available on request.
