# Supplementary material for: Pathways to wheat self-sufficiency in Africa
Source: Glob Food Sec. 2023 Jun;37:100684. doi: 10.1016/j.gfs.2023.100684 (PMC10282895; doi:10.1016/j.gfs.2023.100684)
Supplement: Multimedia component 1 [file mmc1.docx]

**Supplementary Table 1. Changes in wheat area, production, and yield in response to the world commodity price shock in 2008 across major wheat producing countries in Africa.** **%∆ refers to the percentage change between 2008 and 2009. Source: FAOSTAT.**

| **Region** | **Country** | **Harvested area**  **(Mha)** | | |  | **Production**  **(Mt)** | | |  | **Yield**  **(t ha^-1^)** | | |  | **Consumption**  **(kg capita^-1^)** | | | |
| --- | --- | --- | --- | --- | --- | --- | --- | --- | --- | --- | --- | --- | --- | --- | --- | --- | --- |
|  |  | **2008** | **2009** | **%∆** |  | **2008** | **2009** | **%∆** |  | **2008** | **2009** | **%∆** |  | **2008** | **2009** | **%∆** | **%∆ energy*** |
| Northern Africa | Algeria | 1.01 | 1.85 | **83.7** |  | 1.11 | 2.95 | **165.8** |  | 1.1 | 1.6 | **44.7** |  | 183.5 | 189.1 | 3.0 | 3.5 |
|  | Egypt | 1.23 | 1.34 | **8.9** |  | 7.98 | 8.52 | **6.8** |  | 6.5 | 6.4 | -1.8 |  | 145.6 | 145.1 | -0.3 | -0.8 |
|  | Morocco | 2.86 | 2.98 | **4.2** |  | 3.77 | 6.37 | **69** |  | 1.3 | 2.1 | **62.2** |  | 177.3 | 177.4 | 0.0 | 1.3 |
|  | Tunisia | 0.54 | 0.79 | **46.9** |  | 0.92 | 1.65 | **79.9** |  | 1.7 | 2.1 | **22.5** |  | 194.2 | 203.9 | 5.0 | 0.8 |
| Eastern Africa | Ethiopia | 1.42 | 1.68 | **18.2** |  | 2.31 | 3.08 | **32.9** |  | 1.6 | 1.8 | **12.5** |  | 33.2 | 36.3 | 9.3 | 3.4 |
|  | Kenya | 0.13 | 0.13 | **1.0** |  | 0.34 | 0.22 | -34.9 |  | 2.6 | 1.7 | -35.5 |  | 22.5 | 24.8 | 9.9 | 8.7 |
|  | Tanzania | 0.04 | 0.15 | **245.7** |  | 0.04 | 0.08 | **90** |  | 1 | 0.6 | -45 |  | 12.7 | 17.6 | **38.7** | **43.7** |
| Southern Africa | South Africa | 0.75 | 0.64 | -14.1 |  | 2.13 | 1.96 | -8.1 |  | 2.8 | 3.1 | 7.8 |  | 60.1 | 59.5 | -1.0 | -0.1 |
|  | Zambia | 0.01 | 0.03 | **201** |  | 0.11 | 0.2 | **72.6** |  | 9.9 | 5.7 | -42.7 |  | 12.5 | 12.2 | -2.3 | -3.4 |

** %∆ energy refers to the* *share of wheat as source of energy in cereals in kcal/capita/year.*

**Supplementary Table 2. Current area of different crop types per country in areas classified with ‘excellent’ suitability for wheat production in Figure 7. Abbreviations: RTB = roots, tuber, and bananas. All calculations were done based on current cropland, so there was no need to exclude protected areas and forests. All data have a unit of 1000 ha.**

|  |  | **MapSPAM** | | | | | | |
| --- | --- | --- | --- | --- | --- | --- | --- | --- |
|  |  | **Current cropland** | **Suitable cropland** | **Suitable**  **wheat** | **Suitable other cereals** | **Suitable legumes** | **Suitable RTBs** | **Suitable**  **commodities** |
| Ethiopia |  | 8274.8 | 6531.8 | 1180.1 | 4202.1 | 534.0 | 119.6 | 496.0 |
| Tanzania |  | 10826.5 | 6413.7 | 67.3 | 2958.3 | 1171.4 | 1679.7 | 537.0 |
| Zambia |  | 2137.7 | 1535.5 | 26.4 | 906.7 | 251.2 | 160.7 | 190.5 |
| South Africa |  | 4238.2 | 642.6 | 27.5 | 327.3 | 77.4 | 15.0 | 195.4 |
| Madagascar |  | 2553.4 | 987.5 | 2.0 | 557.4 | 59.1 | 270.5 | 98.5 |
| Uganda |  | 6833.9 | 4186.0 | 10.0 | 1209.6 | 1074.2 | 1473.7 | 418.5 |
| Angola |  | 4395.6 | 1538.9 | 2.2 | 854.7 | 433.9 | 239.5 | 8.6 |
| Zimbabwe |  | 2890.4 | 1238.2 | 4.4 | 793.2 | 188.7 | 43.8 | 208.1 |
| Kenya |  | 4705.2 | 3320.4 | 71.3 | 1742.5 | 835.9 | 277.2 | 393.5 |
| DR Congo |  | 5756.8 | 1252.7 | 7.7 | 438.8 | 256.3 | 498.6 | 51.3 |
| Malawi |  | 3854.7 | 2295.9 | 2.5 | 1173.2 | 626.2 | 316.8 | 177.2 |
| Mozambique |  | 5903.3 | 625.2 | 5.6 | 335.6 | 126.4 | 120.5 | 37.1 |
| Burundi |  | 1328.2 | 1253.3 | 13.7 | 250.7 | 294.2 | 617.0 | 77.7 |
| Rwanda |  | 1808.7 | 1739.8 | 44.9 | 358.5 | 447.3 | 832.2 | 56.9 |
| Cameroon |  | 4762.5 | 609.0 | 0.6 | 192.5 | 209.7 | 139.0 | 67.2 |
| Nigeria |  | 28960.2 | 583.6 | 0.0 | 338.9 | 104.6 | 136.3 | 3.8 |
| Swaziland |  | 153.1 | 68.3 | 0.3 | 46.6 | 7.8 | 4.3 | 9.3 |
| Eq. Guinea |  | 2679.5 | 126.3 | 0.0 | 91.3 | 10.3 | 19.8 | 4.9 |
| **Total** |  | **102062.7** | **34948.7** | **1466.5** | **16777.9** | **6708.6** | **6964.2** | **3031.5** |


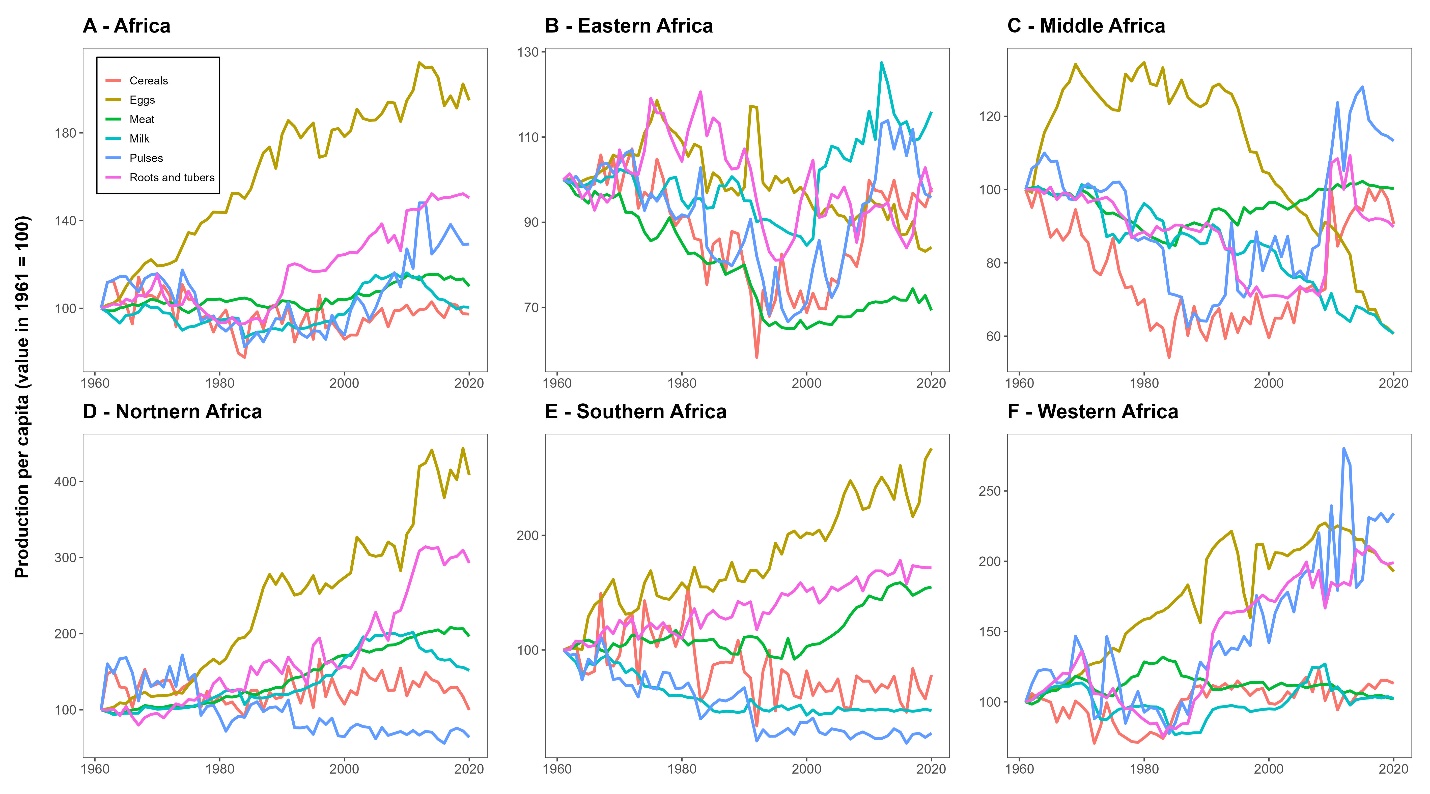


**Supplementary Figure 1. Production per capita of major food groups (cereals, eggs, meat, milk, pulses, and roots and tubers) from 1961 to 2020 in Africa and per region of Africa.**
